# The interactions of human ovarian cancer cells and nanotextured surfaces: cell attachment, viability and apoptosis studies†

**DOI:** 10.1039/c9ra03783g

**Published:** 2019-08-19

**Authors:** Gökçen Yaşayan, Oya Orun, Pınar Mega Tiber, Veronika Rožman, Sevgi Koçyiğit Sevinç

**Affiliations:** Department of Pharmaceutical Technology, Faculty of Pharmacy, Marmara University İstanbul 34668 Turkey gokcen.yasayan@marmara.edu.tr; Department of Biophysics, Faculty of Medicine, Marmara University İstanbul 34854 Turkey; Faculty of Pharmacy, University of Ljubljana 1000 Ljubljana Slovenia

## Abstract

Understanding cell responses to the topography they are interacting with has a key role in designing surfaces due to the distinctiveness in the responses of different cell types. Thus far, a variety of surface textures have been fabricated, and the cellular responses of diversified cell lines to the surface textures have been assessed together with surface chemistry. However, the results reported in the literature are contradictory, and also not in-depth for inferring the relevance between cells, surface chemistry, and surface topography. Starting from this point of view, we focused on fabricating surfaces having extracellular matrix-like surface patterns and investigated the influence of patterning on human ovarian cancer cells. In this study, hemispherical protrusion-shaped, nanotextured surfaces were prepared *via* colloidal lithography and polymer casting methods using monolayer templates prepared from 280 nm, 210 nm, and 99 nm polystyrene particles and polydimethylsiloxane moulds. Then, the surface textures were transferred to biocompatible polycaprolactone films. After the characterisation of the surfaces *via* atomic force microscopy, X-ray photoelectron spectroscopy, and contact angle measurements, the cellular response to topography was evaluated by cell attachment, viability, and apoptosis studies. The results were compared with non-textured surfaces and control plate wells. The results showed that human ovarian cancer cell attachment increased with nanotexturing, which suggests that nanotexturing may be a promising approach for cancer cell modulation, and may have the potential to introduce new strategies for cancer treatment.

## Abstract 2

Fabrication and characterisation studies of nanotextured polycaprolactone surfaces, and an investigation of their influence on human ovarian carcinoma cells.

## Graphical abstract

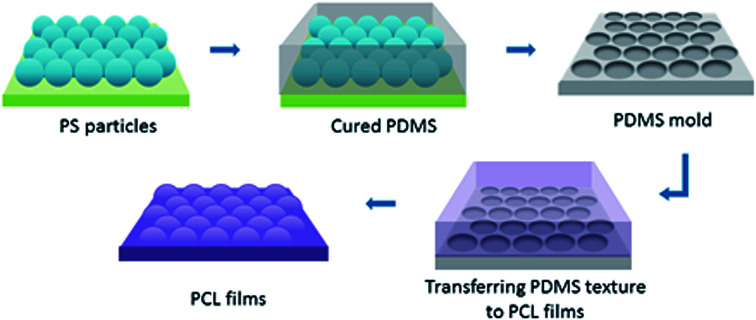

## Introduction

Extracellular matrix (ECM) is a complex meshwork that enables natural cell support and guidance due to its structural, functional, and compositional properties. The structural properties include the ECM texture. Native ECM texture is mostly formed of protrusions, grooves, pits, and pores. Its texture can vary with regions in the body and by developmental stages, which creates a specific micro- and nano-patterned environment composed of various geometries for the cells that are interacting.^1–6^ For instance, nanopits appear in the aortic valve in the heart and in the basement membrane of the cornea, while nanogrooves are in vasculature basement membranes and tendon tissues.^7,8^ Nanoprotrusions have been observed in a large number of tissues.^9,10^ Cell–ECM interactions are important in determining cell behaviour, along with cell–cell interactions. Studies have demonstrated that ECM topography has a role in cellular guidance. It is well-known that a number of cellular reactions are triggered, including migration, proliferation, differentiation, activation of intercellular signalling pathways, induction of transcription factors, and gene expression.^11,12^

Due to the ECM's regulation potential of cell behaviour, the use of engineered surfaces that mimic the functions of ECM, and thus governing cellular mechanisms is a novel area of research providing versatile opportunities.^13^ Among ECM-mimicking designs, textured surfaces provide a wide variety of surface patterns as cell binding sites. These patterns can be regular or irregular, which include diverse geometries, such as grooves, wells, pits, and protrusions of different dimensions in various scales.^14,15^

In literature, it is reported that surface textures can influence the cell behaviour in different ways depending on the surface topography and cell type. Studies have shown that cells respond to surface features on nano- and micro-scale, and changes in cell morphology, adhesion, cytoskeletal arrangement, migration, proliferation, differentiation, and gene expression can be observed.^7,16–20^ It has been demonstrated that not only the geometry of the surface features, but other parameters, such as the distances between features, their aspect ratio, nanoscale symmetry and disorder, are also crucial for cellular responses.^21–26^ Cell type is another fundamental factor. Owing to the fact that cells are programmed to react to the specific structure of ECM, certain cell lines are more sensitive to specific texture geometries.^14^ Although studies have been carried out with various surface geometries and different cell lines, the relationship between pattern geometries and cell lines, and the intrinsic cellular mechanisms triggered due to interactions still remain unclear.^19,23^

In this study, we focused on a better understanding of the relationship amongst surface textures, surface chemistry, and cellular responses. With this aim, large-scale and defect-free hemispherical protrusion shaped nanotextured surfaces were fabricated and characterised in a similar manner to our previous publication.^27^ To transfer texture morphology, an FDA-approved polymer, polycaprolactone (PCL), was selected for film preparation. PCL has gained a lot of interest within numerous diverse areas due to its biodegradable and biocompatible properties, in addition to its low cost.^28^ The high toughness, superior mechanical properties, and slow degradation rate of PCL make it a favourable polymer to ensure the durability of the surface texture for a long period of time.^29^ After the fabrication of the nanotextured PCL films, the films were evaluated by cell culture studies. It is generally reported that human cancer cells are less adhesive compared to other types of cells, which allows for their migration in the body, and also leads to the characteristic morphologies of tumours identified by histological structure destruction.^30,31^ A cancer cell line is selected for further studies based upon the modulation such as increasing adhesion of cancer cells by textured surfaces may offer new possibilities to develop new strategies for the treatment of cancer. We particularly selected the human ovarian cancer cell line (OVCAR-3) due to two reasons. OVCAR-3 is one of the most aggressive cancer type, yet its behaviour on textured surfaces is lesser known than other cancer cell lines, such as breast cancer cells.^32^ Moreover, in our previous study, OVCAR-3 adhesion was found to increase on nanotextured poly lactic-*co*-glycolic acid (PLGA) films prepared from 280 nm polystyrene beads compared to non-textured surfaces.^27,33^ Therefore, in this study, we aimed to assess the OVCAR-3 response in depth by using various texture geometries.

**Fig. 1:**
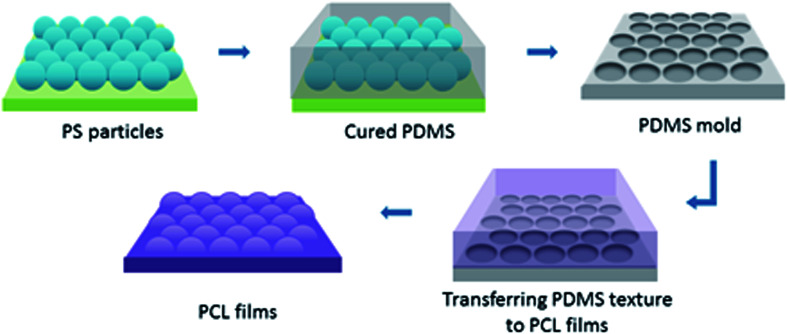
A schematic representation for the nanotextured PCL film fabrication. PS particle topographies were transferred to reverse PDMS moulds, and these moulds were used to obtain the nanotextured PCL films.

To create surface textures, colloidal lithography and polymer casting methods were used on a biodegradable polymer film as described elsewhere.^27,34^ Basically, a self-assembled monolayer of polystyrene (PS) particles was formed at the air–water interface, and then transferred to a silicon wafer for use as a template. For this purpose, polystyrene suspensions with diameters 280 nm, 210 nm and 99 nm were used. Then, the reverse moulds of the PS particle templates were prepared from poly(dimethylsiloxane) (PDMS), which enabled the transfer of topographies of the PS particle templates to film surfaces, and nanotextured films were obtained (Fig. 1). The surfaces were characterised *via* atomic force microscopy (AFM), X-ray photoelectron spectroscopy (XPS), and contact angle measurements. Cellular responses to nanotextured surfaces were studied using the OVCAR-3 cell line. The OVCAR-3 responses to 280 nm, 210 nm and 99 nm nanotextured films were examined by cell attachment, viability, and apoptosis studies, and the results were compared to the data obtained from non-textured surfaces (0 nm).

## Experimental

### Preparation of mono-layer polystyrene (PS) templates

Mono-layer PS templates were prepared following previously described procedure.^27,34,35^ Silicon wafers (Sigma-Aldrich) were cut into square (∼1.5 × 1.5 cm^2^) and rectangular (∼1 × 3 cm^2^) pieces, and sonicated with ethanol. After washing several times with Milli-Q water (Millipore, resistivity of 18.2 MΩ cm^−1^ at 25 °C) and drying at room temperature, the silicon wafers were treated with UV/Ozone for 20 minutes to improve wafer hydrophilicity (UV/Ozone ProCleaner™ Plus, BioForce Nanosciences, Inc.). A Petri dish was filled with Milli-Q water, and the PS particle suspension (100 μl, 10 wt%, Bangs Labs) with diameters 280 nm, 210 nm or 99 nm was mixed with an equal amount of ethanol. This mixture was discharged slowly over the rectangular silicon wafer, which was placed in a Petri dish at a sloped plane to the Milli-Q water surface using a micropipette, and the rectangular wafer was then slowly submerged in the water. A sodium dodecyl sulphate (SDS) solution (2%, ∼10 μl) was added to the Petri dish in order to acquire hexagonal close-packed particles. The PS monolayers on the water layer were then lifted off by a square silicon wafer, and dried at room temperature.

### Preparation of PDMS templates

PS-coated silicon wafers with the diameters 280 nm, 210 nm or 99 nm were placed in a borosilicate glass Petri dish. PDMS (Sylgard 184 silicone elastomer, Dow Corning) was mixed thoroughly in accordance with the manufacturer's instructions, and poured over the borosilicate glass Petri dish. Residual bubbles were removed under vacuum, and the Petri dish was kept at 40 °C for 2 h for the curing procedure. The PDMS template was separated from the PS template and washed with acetone to eliminate the remaining PS particles. 0 nm PDMS templates were prepared without the use of PS beads following the above described procedure.

### Preparation of PCL films

PCL (1 g, average *M*_n_ 45 000, Sigma Aldrich) was dissolved in chloroform (10 ml), and then poured over the PDMS moulds with diameters 280 nm, 210 nm or 99 nm. After solvent evaporation, the PCL films were peeled off from the moulds and washed several times with *n*-hexane to remove the residual PDMS. 0 nm PCL films were prepared using non-textured PDMS moulds following the above described procedure.

### AFM studies

The topography images of the PS, PDMS, and PCL films were obtained in air using an Ambient AFM™ (NanoMagnetics Instruments) operating in a dynamic mode. PPP-NCLR AFM probes (nominal resonance frequency = 190 kHz, nominal force constant = 48 N m^−1^, Nanosensors, Switzerland) were used, and images were acquired using an E-scanner at scan rates ranging from 0.5 to 1.5 Hz. The image data was analysed using an NMI Image Analyzer v1.5 (NanoMagnetics Instruments).

### XPS measurements

The surface elemental composition of the PCL films was examined by X-ray photoelectron spectroscopy (XPS). XPS studies were performed on a PHI 5000 Versa Probe (ULVAC-PHI, Inc.) model X-ray photoelectron spectrometer instrument equipped with a monochromatic Al Kα X-ray source (1486.6 eV) as the X-ray anode at 26 W at a fixed incident angle of 45°. The pressure inside the analyser was maintained at 10^−7^ Pa. The binding energies were calibrated by setting the C 1s peak to 284.5 eV as a reference. Data analysis was performed using the Multipak software. The measurements were performed with 280 nm, 210 nm, 99 nm, and 0 nm PCL films. Samples were washed with *n*-hexane for the removal of residual PDMS before measurements, and were inserted into the spectrometer on glass slides after solvent evaporation.

The residual PDMS layer thickness over the PCL films were calculated from the XPS silicon signal data. Topofactor calculations of the textured films were performed in order to determine the equivalent conformal PDMS thickness over the films.^36,37^

### Water contact angle measurements

The water contact angle of the PCL films were measured using a KSV/Attension THETA Optical Tensiometer (Helsinki, Finland). Contact angle measurements were performed *via* the sessile drop technique by dispersing an ultrapure water droplet with a volume of ∼5 μl. The water contact angles of the droplets were analysed by OneAttension image analysis software Version 2.6. The measurements were performed at a minimum of 3 random locations at room temperature. The measurements were performed with 280 nm, 210 nm, 99 nm, and 0 nm PCL films.

### Cell culture studies

The OVCAR-3 cell line (ATCC HTB-161) was cultivated in an RPMI-1640 medium supplemented with fetal bovine serum (10%, FBS, Sigma, UK), penicillin/streptomycin (100 U ml^−1^ and 100 μg ml^−1^, respectively, Life Tech), bovine insulin (0.01 mg ml^−1^, Life Tech) under standard cell culture conditions with 5% CO_2_ at 37 °C. The films were sterilized by soaking in 75% ethanol for approximately 2 h, following UV sterilization for 30 min in ethanol and later 30 minutes without ethanol. After rinsing with the RPMI-1640 medium (Life Tech), the films were placed in a 24 well plate in 3 ml of RPMI-1640 medium containing 10% FBS. The cells were seeded on films in a 24 well culture plate for cell adhesion, viability and apoptosis studies as explained below. Studies were carried out with the 0 nm PCL films, and the textured PCL films prepared from 280 nm, 210 nm and 99 nm PS templates. For control studies, a control well of a 24 well plate (flat plastic) was used. All cell culture assays were repeated in triplicate at desired time points. Cell number averages and standard deviations were calculated for each time point.

### Cell adhesion assay

Cells were seeded on top of the films with an initial cell plating density of 1 × 10^5^ cells per film in a 24 well culture plate. The media was added and then, the cell cultures were kept for 4 h and 24 h under standard cell culture conditions with 5% CO_2_ at 37 °C. At the end of the incubation times, the non-adherent cells were removed by washing three times with PBS. In order to avoid the contribution of adherence to the well plate plastic, the films with the cells were transferred to a new well plate, trypsinized and the number of cells attached to the films were counted using a hemocytometer after trypan blue staining.

### Cell viability

A 3-(4,5-dimethylthiazol-2-yl)-2,5-diphenyl tetrazolium bromide (MTT) assay was performed to evaluate the viability and cytotoxicity of the cells on the polymer surfaces. Cell viability was determined using the MTT Cell Proliferation Assay Kit (Life Tech). Cells were seeded on films with the initial density of 5 × 10^4^ cells per film in a 24 well culture plate, and incubated for 24 h and 48 h with 5% CO_2_ at 37 °C. At the end of each incubation time, the films were transferred to a new well plate to avoid false contribution from plastic, and the cells were incubated with an MTT labelling reagent for 4 h. A 100 μl aliquot of the solubilisation solution was added to each well and the plate was incubated overnight. The absorbance of the samples was measured at 570 nm using a scanning multiwell spectrophotometer (Synergy H1 multi-mode microplate reader, Biotek).

The cell viability was further assessed after trypan blue staining either by direct counting or using a Tali® image-based cytometer (ThermoFisher), or both. The cells were seeded on films with an initial plate density of 5 × 10^4^ cells per film in a 24 well culture plate, and incubated for 24 h and 48 h. At the end of each incubation time, the films were transferred to a new well plate. The cells were lifted from the films through trypsinization, stained with trypan blue, and counted. Cell viability studies were performed for up to 48 h in order to avoid cell growth confluence.

### Determination of apoptosis

An annexin V/propidium iodide binding assay was performed for the determination of apoptosis. The Tali® Apoptosis kit – Annexin V Alexa Fluor® 488 and propidium iodide (ThermoFisher) was used according to the manufacturer's instructions. Briefly, the cells were plated onto films with an initial density of 5 × 10^4^ cells per film in a 24 well culture plate, and incubated for 24 h and 48 h at standard cell culture conditions. At the end of each incubation time, the films with cells were transferred to a new well plate. Cells were lifted from the films through trypsinization, centrifuged, resuspended in annexin binding buffer and incubated with Annexin V Alexa Fluor® 488 at room temperature in dark for 20 min. Following the centrifugation, the cells were again resuspended in the annexin binding buffer and incubated with propidium iodide at room temperature in the dark for 5 min. A Tali® image-based cytometer (ThermoFisher) was used for the discrimination of apoptotic cells and necrotic cells from live cells in a population, and the number of live, apoptotic and dead cells were calculated.

The mitochondrial membrane potential (MMP) assay was performed as the second method to evaluate apoptosis. Cells were plated on films with an initial density of 5 × 10^4^ cells per film in a 24 well culture plate and incubated for 24 h and 48 h. At the end of each incubation time, the films were transferred to a new well plate. The cells were lifted and incubated with the Mitotracker JC-1 (JC-1 Mitochondrial Membrane Potential Assay Kit, Abnova) for another 15 min. After washing with buffer solution, the fluorescence was measured in dark using the excitation/emission wavelengths at 535/595 nm as the red fluorescent emission (indicating high MMP of healthy cells) and 485/535 nm as the green fluorescent emission (indicating low MMP). The results are expressed as the ratio of green to red fluorescence intensity.

### Statistical analysis

A *t*-test was performed to determine statistical significance. Results were considered statistically significant at *p* < 0.05.

## Results and discussion

Developing novel biomaterials that can alter the behaviours of living organisms during their interactions is a growing area of interest, especially over the last decades. Among these biomaterials, textured surfaces draw attention due to their distinctive possibilities of influencing cell responses based on the mimicking ECM topography.^15^ Studies with various topographical geometries with various diameters have reported changes in cell responses on textured surfaces. Besides topography, cell line choice is another significant factor as the cell response to a particular topography can vary depending on the cell line.^16,38^

In our previous study, a novel method was developed to obtain defect-free nanotextured surfaces with regular surface textures over large scales. The cell attachments of OVCAR-3 cells alone and co-cultured with MSCs on the 280 nm nanotextured surfaces of poly(lactic-*co*-glycolic acid) were investigated, and in both cultures, it was observed that cell attachment was increased in nanotextured surfaces after 24 h.^27^

In this study, we aimed to study the behaviour of the OVCAR-3 cells on nanotextured surfaces in depth. In the first step of study, hemispherical protrusion-shaped nanotextured surfaces were prepared using the method reported in the previous study. Polystyrene particles were used to create surface textures with diameters 280 nm, 210 nm, and 99 nm. We have selected PCL to transfer the surface morphologies to fabricate biocompatible, biodegradable, and durable surfaces. After fabrication, surfaces were characterised and the influence of surface textures on OVCAR-3 cells were evaluated.

### Preparation and AFM studies of nanotextured surfaces

Nanotextured surfaces were fabricated using nanosphere lithography with the method described previously.^27,34,35^ Briefly, hexagonally packed monolayer PS particle arrays were formed on the air–water interface and transferred to a silicon wafer. This wafer was used to produce reverse PDMS moulds, by which the topography of PS arrays was transferred to the PCL films. Three different PS particle diameters (280 nm, 210 nm, and 99 nm) were used to produce PS wafers. Non-textured flat surfaces, referred to as 0 nm, were fabricated with the same procedure without the use of PS beads and were used for control studies (Fig. S2, ESI†).

**Fig. 2:**
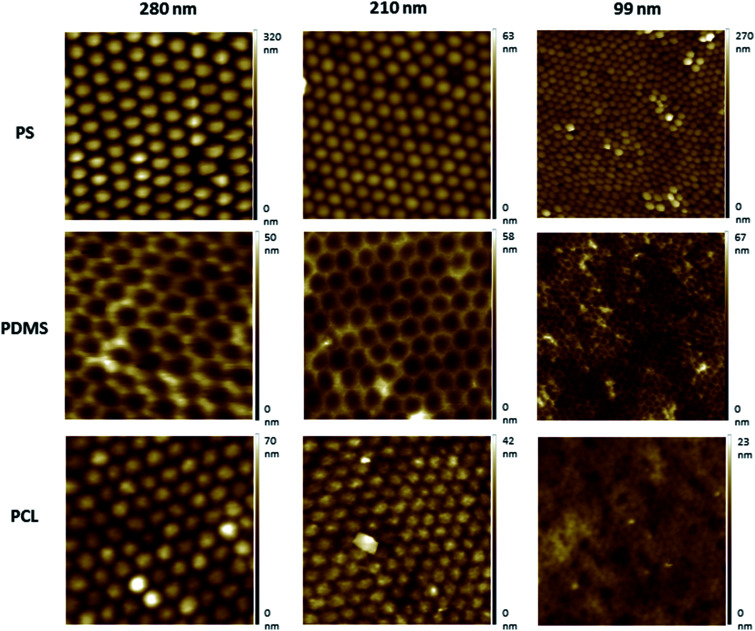
AFM topography images of the nanotextured surfaces fabricated using the nanosphere lithography. 280 nm, 210 nm, and 99 nm PS particles were used for the preparation of PS surfaces. The PS surface topography was transferred to PDMS moulds and PCL surfaces. All images were 2 μm × 2 μm scans.

AFM images indicated that the topographies of the monolayer PS arrays were successfully transferred to the PDMS moulds and PCL films for the templates prepared by using 280 nm and 210 nm PS particles, and the hexagonally packed ordered arrays were obtained without structural defects (Fig. 2). For the PS wafers prepared from the 99 nm particles, PS clusters were observed during AFM imaging. Surface disruption was probably obtained during lifting the wafer off the air–water interface as a result of smaller diameter (99 nm) of PS particles compared to other particles. These PS clusters could not be prevented despite varying the percentage of SDS solution and carefully lifting the wafer off the air/water interface during template preparation, as congruently published elsewhere.^27^ The surface defects were transferred to the PDMS templates and PCL films.

Protrusion heights were measured from the AFM image data (Fig. S1, ESI†). The height values were found to be of around 22 nm for the surfaces prepared using the 280 nm PS particles, and ∼13 nm for the 210 nm PS particles. Due to the surface disruption of the 99 nm surfaces, the height values were found to vary between ∼2–15 nm, and the presence of pits were also observed.

Nanotopography can increase the surface energy compared to flat surfaces. It is reported that even small changes in the surface roughness can significantly increase the surface energy, and the nano-scale surface roughness can promote the cellular attachment and growth.^39,40^ Surface roughness values were calculated from the AFM image data, and it was found to be 10 nm for the nanotextured surfaces (280 nm, 210 nm and 99 nm surfaces), and 0 nm for the 0 nm surfaces. Although there is no difference in the surface roughness amongst the nanotextured surfaces, the increase compared to flat surfaces is favoured due to its possible impact for increasing the cellular adhesion.

### XPS and water contact angle measurements

XPS studies were performed on textured and 0 nm samples as well as PCL pellets to determine the amount of PDMS remaining on the PCL film surfaces. PDMS is the most widely used material to prepare elastomeric moulds for pattern transferring. It exhibits ideal properties for replica moulding in soft lithography, mainly because of its transparency, non-toxicity, biocompatibility, and elastomeric properties.^41^ However, as a drawback of its use, PDMS may be transferred over the underlying sample as a residual layer. Also, the overlay PDMS thickness can vary over the underlying sample, related to the density and layout of the pattern to be transferred. Owing to the fact that the uniformity of the residual layer thickness all over the sample is an indicator of pattern transfer uniformity,^42^ the PDMS thickness over the underlying sample was calculated by XPS studies.

According to data, it was found that both textured and 0 nm films have PDMS residuals. From the data acquired, the PDMS thickness over the PCL films were calculated using the method described previously.^36^ By applying the XPS topofactor, the overlayer PDMS thicknesses on the topographic surfaces were calculated by converting equivalent planar thickness to the actual PDMS thickness.^36,37^ After the topofactor calculations, it was found that all nanotextured samples have similar PDMS thickness values over the films; all topofactor corrected textured films have ∼3 nm PDMS remaining, which is approximately the same value with non-corrected 0 nm films (Table 1). A two sample *t*-test was performed, and it was found that there was no statistical significance between the textured and 0 nm films (*p* > 0.05).

**Table 1:** XPS results, PDMS thickness values, and contact angle measurements of the samples. All studies were carried out with nanotextured films (280 nm, 210 nm, and 99 nm) and 0 nm films. Non-corrected and topofactor-corrected PDMS thickness values were calculated from the XPS results.^36,37^ Control studies for XPS were carried out using PCL pellets

Samples	XPS results			PDMS thickness (nm)		Contact angle (*θ*)
	C 1s, %	O 1s, %	Si 2p, %	Non-corrected	Topofactor corrected	
280 nm	56.6 ± 0.38	27.8 ± 0.35	15.5 ± 0.57	3.9 ± 0.24	2.8 ± 0.17	89.2 ± 0.53
210 nm	54.7 ± 1.91	28.1 ± 0.70	17.2 ± 1.35	4.7 ± 0.72	3.4 ± 0.52	89.8 ± 5.81
99 nm	55.1 ± 1.25	28.5 ± 0.31	16.5 ± 1.19	4.3 ± 0.58	3.1 ± 0.42	86.8 ± 1.12
0 nm	57.2 ± 0.15	28.4 ± 0.21	14 ± 0.42	3.3 ± 0.15	—	86.5 ± 0.63
PCL pellets	76.1 ± 0.58	23.7 ± 0.5	—	—	—	—

Contact angle measurements demonstrated that the surface texture did not change the surface wettability. Similar wettability patterns were obtained for both textured and 0 nm surfaces. The contact angle values of the samples were found to be between 87–90°, which indicates the moderate wetting properties of samples.

### Cell culture studies

Adhesion, viability, and apoptosis studies were performed by culturing the OVCAR-3 cells on the nanotextured (280 nm, 210 nm, and 99 nm) and 0 nm films. A flat plate well was used for control studies.

### Human ovarian carcinoma cell adhesion assay

**Fig. 3:**
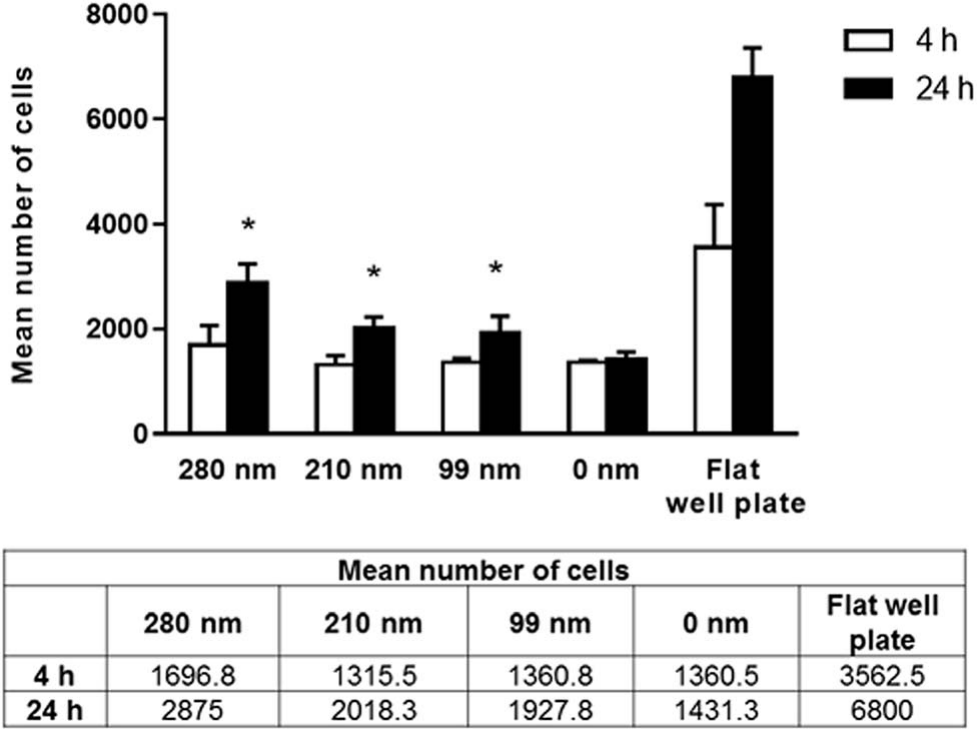
Mean number of adhered cells on nanotextured (280 nm, 210 nm, and 99 nm) and 0 nm films, and flat well plate on 4^th^ hour and 24^th^ hour. Studies were carried out with the OVCAR-3 cell line (*p < 0.05 for the comparison with 0 nm films (control group)).

Cells require specific surface attachments. When cells cannot interact with the substrate appropriately, they detach from the surface and the cell death triggered by this mechanism is called anoikis.^43^ In our study, we performed adhesion studies first to see if cell attachment could occur between the cells and nanotextured surfaces. In the adhesion studies, the mean number of adhered cells to the surfaces after 4 and 24 h of incubation were evaluated, and the results are given in Fig. 3. According to our adhesion studies, the flat plate well has the highest number of cell attachment both for 4 h and 24 h. After 4 hours of cell adhesion, the number of cells on the film surfaces was similar for the 210 nm and 99 nm nanotextured surfaces and the 0 nm surfaces. For the 280 nm surfaces, the mean number of cells was found to be higher compared to that of other surfaces. At 24 h, the number of cells on surfaces were increased, and cell adherences to the nanotextured surfaces of all diameters were higher compared to 0 nm surface. Similarly, the 280 nm surfaces have the highest number of cell attachment at 24 h compared to other film surfaces, and the increase in the cell attachment on nanotextured films (280 nm, 210 nm and 99 nm) were found to be statistically significant compared to the 0 nm films (*p* < 0.05).

### Human ovarian carcinoma cell viability assay

**Fig. 4:**
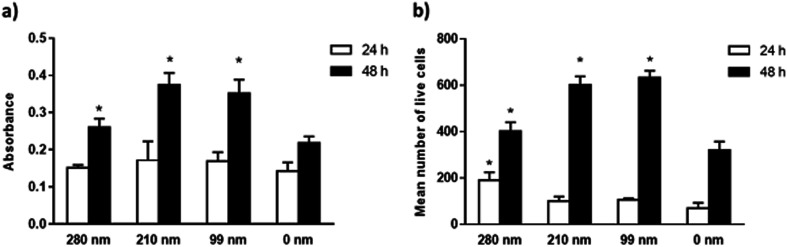
Viability studies of the OVCAR-3 cells on nanotextured and 0 nm films. Cells were seeded on PCL films for 24 h and 48 h, and cell viability studies were carried out by the MTT assay (a), and direct cell count after trypan blue staining (b), as described in methods. The results are expressed as the mean ± SD for each group (*p < 0.05 for the comparison with 0 nm films).

The viabilities of the OVCAR-3 cells at 24 and 48 h of incubation were evaluated on nanotextured and 0 nm films *via* the MTT assay. Results are given in Fig. 4. In the MTT assay, a colorimetric reaction was followed to assess cell metabolic activity that represents the number of viable cells. The change in cell numbers was also evaluated by direct cell counts after trypan blue staining. The data obtained in both assays were found to have similar trends. In both assays, it was observed that the cell viability increased with time for all films. However, the nanotextured surfaces promoted cell viability better than 0 nm surfaces. It was found that the well plate control sample has the highest cell viability at both time points (Fig. S3, ESI†). MTT studies indicated that the absorbance values were similar for all films at 24 h. At 48 h, the absorbance values increased much more on the nanotextured films as compared to that on 0 nm films, and these increases were statistically significant compared to 0 nm (*p* < 0.05) (Fig. 4a). The 210 nm and 99 nm films were found to have greater absorbance values compared to the 280 nm nanotextured surfaces at 48 h.

In the trypan blue staining assay, the trend was similar but the increase in the cell number was sharper compared to the MTT assay at the end of 48 h. Results indicated a time-dependent increase in the live cell attachment to smaller surface diameters. According to results, the live cell number was higher on the 280 nm surface diameters after 24 h of interactions in a similar fashion to the cell adhesion studies. However, the number of live cells was higher at 48 h on the 210 nm and 99 nm surfaces compared to that on the 280 nm surfaces. In comparison to 0 nm surfaces, the mean number of live cells was found to be higher in the nanotextured surfaces, and the increase in the number of live cells was found to be statistically significant for the 280 nm films at 24 h, and for 280 nm, 210 nm and 99 films at 48 h compared to that for 0 nm surfaces (*p* < 0.05) (Fig. 4b).

Although cells respond both to nanotopography and microtopography, they are in contact with nanotopographical features in their natural microenvironment. A basement membrane composed of a complex topography consists of protrusions, pores, and fibres in the range of 30–400 nm.^1^ It is often reported that the cellular response increases in correlation with a decrease in the topography diameters, probably owing to the well-developed lamellipodia and filopodia formation that enhances cellular attachment, and thereby encourages cell spreading.^44^ Cells sense the surrounding environment by these extensions, and the interactions among filopodia and topography could be crucial to determining the cellular response to topography. Dalby *et al.* have studied fibroblast filopodia interactions with 10 nm high nano-islands, 27 nm high nano-islands, and 35 nm nano-pits.^45–47^ In another study, human foetal osteoblastic cell responses were investigated on a nano-island topography of 11 nm, 38 nm, and 85 nm, and the cell adhesion and proliferation were found to be higher in correlation with a decrease in island height.^44^ 70 nm is reported to be a threshold for the protrusion height, where above this protrusion height, the formation of focal adhesions are reduced due to changes in integrin clustering.^7^

In our results, the AFM protrusion height values of all fabricated nanotextured films were found to be below 70 nm, which induced the increased cell adhesion and viability probably by promoting focal adhesion formation in comparison to the non-textured surfaces (Fig. S1, ESI†). The protrusion height values of the surface features did not decrease in correlation with the PS particle diameters due to the disruption of 99 nm particles during preparation. This resulted due to the presence of bilayers and pits on the 99 nm surface, and the height of the features were consequently varied in the range of ∼2–15 nm. For the 280 nm and 210 nm surfaces, ordered monolayers were obtained and the height values of the features decreased in accordance with the PS particle diameter to ∼22 nm and ∼13 nm, respectively. When the cell viability results were evaluated together with the protrusion heights of the features, it is observed that the cell viability was increased in parallel with a decrease in the protrusion height over time. There is no significant difference between the 210 nm and 99 nm surfaces, probably because of having similar feature heights.

### Human ovarian carcinoma cell apoptosis assay

**Fig. 5:**
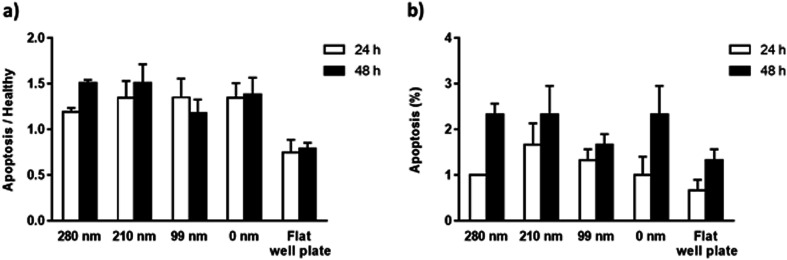
Apoptosis of OVCAR-3 cells on nanotextured and 0 nm films after 24 h and 48 h. MMP assay (a) and annexin V/propidium iodide binding assay (b) were carried. For the MMP assay, cells were stained using a JC-1 dye, and the results are expressed as the ratio of green (indicating apoptotic cells) to red (indicating healthy cells) fluorescence intensity.

The mitochondrial membrane potential (MMP) assay and annexin V/propidium iodide binding assay were performed for the evaluation of apoptosis on the surfaces at the 24^th^ h and 48^th^ h. Mitochondrial dysfunction has been considered as the determinative of apoptotic pathway, and a decrease in MMP is observed during the apoptosis. The cationic dye JC-1 (5,5′,6,6′-tetrachloro-1,1′,3,3′-tetraethylbenzimidazolylcarbocyanine iodide) monomers form aggregates in the mitochondrial matrix, and the aggregates emit red fluorescence. However, when MMP is decreased, JC-1 cannot aggregate in the matrix and the dye monomers emit green fluorescence. When the green/red fluorescence intensity ratios of the nanotextured films were compared with the non-textured surfaces and control well plate, the results did not indicate any significant difference between the samples, suggesting that the nanotextured and 0 nm films are not apoptotic (Fig. 5a).^48,49^

In the annexin V/propidium iodide binding assay, the cell population was counted and the apoptotic cells percentages were calculated for textured films, 0 nm films, and well plate control. According to the results, the percentages of the apoptotic cells for the surfaces were around 1–1.5% for 24 h, and 1.5–2.5% for 48 h (Fig. 5b). When a *t*-test was applied to the results, no significant difference was found compared with the 0 nm surfaces and control well plate, suggesting again that the surfaces are not apoptotic.

## Conclusions

Modulating material–cell interactions has a key importance in designing smart and biofunctional materials for versatile purposes. The formation of topographies on material surfaces is considered one of the tools for achieving that purpose. Although there are numerous studies on the fabrication of surface topographies and their influences on cell behaviour, the link between surface texture and cell lines is not clear yet.

In this project, we aimed at thoroughly gaining insight into the cell–surface interactions, and we have investigated the relationship between hemispherical protrusion shape texture geometries and a cancer cell line, specifically human ovarian cancer cell line by cell attachment, viability and apoptosis studies. In our former study,^27^ we focused on the development of a new method to template hemispherical protrusion-shaped nanotexture on PLGA surfaces adapted from Ogaki *et al.*^34^ By this method, defect-free monolayer PS templates were fabricated on a large scale, and the non-ordered surface topography such as the presence of step edges and macroscopic features were eliminated. After fabrication, preliminary cell attachment was evaluated using the 280 nm nanotextured surfaces only. In this study, cell culture studies were diversified in order to evaluate the potential of the nanotextured surfaces in terms of modulating the OVCAR-3 responses in depth. We studied cell attachment, viability, and apoptosis on the 280 nm, 210 nm, and 99 nm surfaces, as well as on non-textured surfaces and well plate controls.

According to our results, the texture topographies were successfully transferred to PCL films with higher accuracy for the surfaces prepared using the 210 nm and 280 nm PS particles in compared to that using 99 nm particles, without any topographical disruptions. Irregular surfaces were obtained with 99 nm particles, which were characterised with random multi-layers. In the literature, it is reported that the cell–substrate interactions are influenced by various factors, including surface chemistry, roughness, wettability, and the elasticity/stiffness of substrates.^30^ The cell line is another crucial factor. In a prior study, it was found that fibroblasts adhered better to substrates with smaller roughness ratios, independently from the surface wettability and polymer choice, whereas neuronal cells adhered to textured surfaces independent of the surface wettability and roughness.^50^ In order to investigate the influence of topography to the OVCAR-3 cell line behaviour only, the films were characterised thoroughly to ensure that other factors apart from topography possess similar characteristics. According to our results, the topofactor-corrected residual PDMS thickness values, surface roughness and surface wettability properties were found to have similar values for all nanotextured samples.

The cell culture results indicated that the surface texture has an impact on the OVCAR-3 cell behaviour in terms of increased attachment and viability compared to the 0 nm surfaces. A time-dependent increase in the cellular viabilities on smaller hemispherical protrusion diameters were observed, and the 210 nm and 99 nm surfaces were found to be better in terms of the increase in cell viability. This is probably due to similar protrusion heights of 210 nm and 99 nm surfaces as a result of the topographical irregularities of the 99 nm features, and the similar height of features may induce better lamellipodia and filopodia formation of cells compared to 280 nm surface. The apoptosis assays indicated that the nanotextured and 0 nm films are not apoptotic.

We hope the results acquired in this study will be useful in understanding the influence of nanotextured materials on human ovarian cancer cells, and may offer novel possibilities for cancer treatment.

## Conflicts of interest

There are no conflicts to declare.

## Supplementary Material

RA-009-C9RA03783G-s001

## References

[cit1] Abrams G. A., Goodman S. L., Nealey P. F., Franco M., Murphy C. J. (2000). Cell Tissue Res..

[cit2] Abrams G. A., Murphy C. J., Wang Z. Y., Nealey P. F., Bjorling D. E. (2003). Urol. Res..

[cit3] Rosso F., Giordano A., Barbarisi M., Barbarisi A. (2004). J. Cell. Physiol..

[cit4] Hynes R. O. (2009). Science.

[cit5] Hynes R. O. (2014). Nat. Rev. Mol. Cell Biol..

[cit6] Wolf K., Friedl P. (2011). Trends Cell Biol..

[cit7] Biggs M. J. P., Richards R. G., Dalby M. J. (2010). Nanomedicine.

[cit8] Liliensiek S. J., Nealey P., Murphy C. J. (2009). Tissue Eng., Part A.

[cit9] Kim D. H., Provenzano P. P., Smith C. L., Levchenko A. (2012). J. Cell Biol..

[cit10] Janson I. A., Putnam A. J. (2015). J. Biomed. Mater. Res., Part A.

[cit11] Stevens M. M., George J. H. (2005). Science.

[cit12] Kelleher C. M., Vacanti J. P. (2010). J. R. Soc., Interface.

[cit13] Donnelly H., Dalby M. J., Salmeron-Sanchez M., Sweeten P. E. (2018). Nanomedicine.

[cit14] Aminuddin N. I., Ahmad R., Akbar S. A., Pingguan-Murphy B. (2016). Sci. Technol. Adv. Mater..

[cit15] Sousa M. P., Caridade S. G., Mano J. F. (2017). Adv. Healthcare Mater..

[cit16] Martinez E., Engel E., Planell J. A., Samitier J. (2009). Ann. Anat..

[cit17] Teixeira A. I., Abrams G. A., Bertics P. J., Murphy C. J., Nealey P. F. (2003). J. Cell Sci..

[cit18] Lee L. C. Y., Gadegaard N., de Andres M. C., Turner L. A., Burgess K. V., Yarwood S. J., Wells J., Salmeron-Sanchez M., Meek D., Oreffo R. O. C., Dalby M. J. (2017). Biomaterials.

[cit19] Ermis M., Antmen E., Hasirci V. (2018). Bioact. Mater..

[cit20] Nikkhah M., Edalat F., Manoucheri S., Khademhosseini A. (2012). Biomaterials.

[cit21] Dalby M. J., Gadegaard N., Tare R., Andar A., Riehle M. O., Herzyk P., Wilkinson C. D. W., Oreffo R. O. C. (2007). Nat. Mater..

[cit22] Loesberg W. A., te Riet J., van Delft F., Schon P., Figdor C. G., Speller S., van Loon J., Walboomers X. F., Jansen J. A. (2007). Biomaterials.

[cit23] Dalby M. J., Gadegaard N., Oreffo R. O. C. (2014). Nat. Mater..

[cit24] Lehnert D., Wehrle-Haller B., David C., Weiland U., Ballestrem C., Imhof B. A., Bastmeyer M. (2004). J. Cell Sci..

[cit25] Allan C., Ker A., Smith C. A., Tsimbouri P. M., Borsoi J., O'Neill S., Gadegaard N., Dalby M. J., Meek R. M. D. (2018). J. Tissue Eng..

[cit26] Ramirez-San Juan G. R., Oakes P. W., Gardel M. L. (2017). Mol. Biol. Cell.

[cit27] Yasayan G., Xue X., Collier P., Clarke P., Alexander M. R., Marlow M. (2016). Nanotechnology.

[cit28] Woodruff M. A., Hutmacher D. W. (2010). Prog. Polym. Sci..

[cit29] Abedalwafa M., Wang F. J., Wang L., Li C. J. (2013). Rev. Adv. Mater. Sci..

[cit30] Ferrari M., Cirisano F. (2019). Colloids Interfaces.

[cit31] Hirohashi S., Kanai Y. (2003). Cancer Sci..

[cit32] Zhang L., Webster T. J. (2012). Nanotechnology.

[cit33] Mitra A. K., Davis D. A., Tomar S., Roy L., Gurler H., Xie J., Lantvit D. D., Cardenas H., Fang F., Liu Y. Y., Loughran E., Yang J., Stack M. S., Emerson R. E., Dahl K. D. C., Barbolina M. V., Nephew K. P., Matei D., Burdette J. E. (2015). Gynecol. Oncol..

[cit34] Ogaki R., Lyckegaard F., Kingshott P. (2010). ChemPhysChem.

[cit35] Rybczynski J., Ebels U., Giersig M. (2003). Colloids Surf., A.

[cit36] Shard A. G., Wang J., Spencer S. J. (2009). Surf. Interface Anal..

[cit37] Shard A. G. (2012). J. Phys. Chem. C.

[cit38] Kim H. N., Jiao A., Hwang N. S., Kim M. S., Kang D. H., Kim D. H., Suh K. Y. (2013). Adv. Drug Delivery Rev..

[cit39] Chung T. W., Liu D. Z., Wang S. Y., Wang S. S. (2003). Biomaterials.

[cit40] Lampin M., WarocquierClerout R., Legris C., Degrange M., SigotLuizard M. F. (1997). J. Biomed. Mater. Res..

[cit41] Lima M. J., Correlo V. M., Reis R. L. (2014). Mater. Sci. Eng., C.

[cit42] Gates B. D., Xu Q. B., Stewart M., Ryan D., Willson C. G., Whitesides G. M. (2005). Chem. Rev..

[cit43] Gilmore A. P. (2005). Cell Death Differ..

[cit44] Lim J. Y., Hansen J. C., Siedlecki C. A., Runt J., Donahue H. J. (2005). J. R. Soc., Interface.

[cit45] Dalby M. J., Giannaras D., Riehle M. O., Gadegaard N., Affrossman S., Curtis A. S. G. (2004). Biomaterials.

[cit46] Dalby M. J., Gadegaard N., Riehle M. O., Wilkinson C. D. W., Curtis A. S. G. (2004). Int. J. Biochem. Cell Biol..

[cit47] Dalby M. J., Riehle M. O., Johnstone H., Affrossman S., Curtis A. S. G. (2004). Cell Biol. Int..

[cit48] Gottlieb E., Armour S. M., Harris M. H., Thompson C. B. (2003). Cell Death Differ..

[cit49] Ly J. D., Grubb D. R., Lawen A. (2003). Apoptosis.

[cit50] Koufaki N., Ranella A., Aifantis K. E., Barberoglou M., Psycharakis S., Fotakis C., Stratakis E. (2011). Biofabrication.

