# Supplementary material for: The interactions of human ovarian cancer cells and nanotextured surfaces: cell attachment, viability and apoptosis studies
Source: RSC Adv. 2019 Aug 19;9(45):25957–66. doi: 10.1039/c9ra03783g (PMC9070382; doi:10.1039/c9ra03783g)
Supplement: RA-009-C9RA03783G-s001 [file RA-009-C9RA03783G-s001.pdf]

Electronic Supplementary Material (ESI) for RSC Advances.
This journal is © The Royal Society of Chemistry 2019

Electronic Supplementary Material (ESI)

The interaction of human ovarian cancer cells and nanotextured surfaces: cell attachment, viability and apoptosis studies

Gökçen Yaşayan,[a]* Oya Orun,[b] Pınar Mega Tiber,[b] Veronika Rozman, [ac] and Sevgi Koçyiğit Sevinç [b]

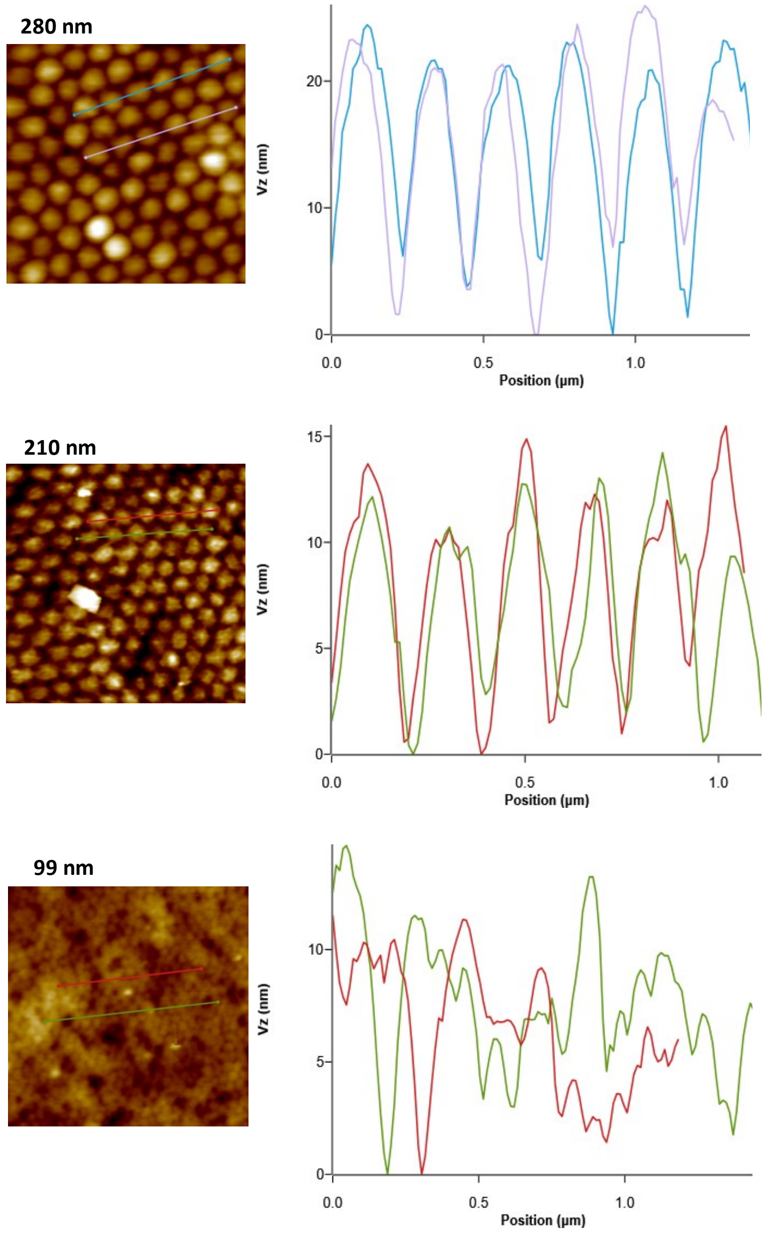

Fig. S1. AFM crossection analysis of nanotextured PCL surfaces fabricated using nanosphere lithography. 280 nm, 210 nm, and 99 nm PS particles were used for preparation of PCL surfaces.

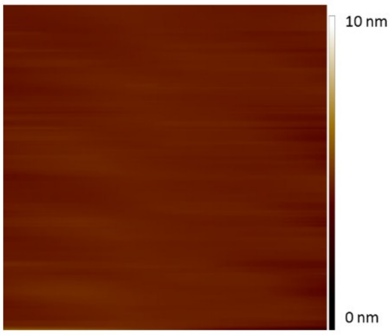

Fig. S2. AFM topography images of non-textured PCL surfaces. Non-textured flat surfaces, referred as 0 nm, were fabricated with the same procedure described without use of PS beads. All images were 2 µm x 2 µm.

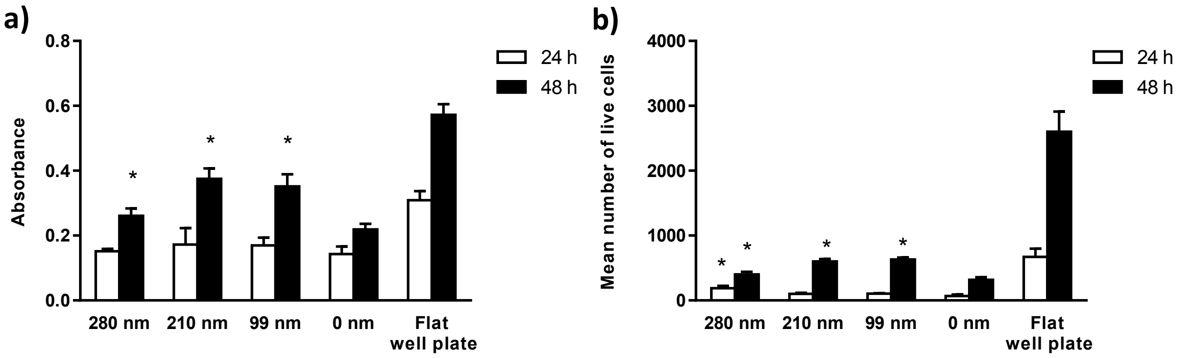

Fig. S3. Viability studies of OVCAR-3 cells on nanotextured films, 0 nm films, and flat well plate. Control studies were carried on flat well plate. Cells were seeded on PCL films for 24 h and 48 h, and cell viability studies were carried by MTT assay (a), and direct cell count after trypan blue staining (b), as described in methods. The results are expressed as the mean ± SD for each group. (* $p < 0.05$ for the comparison with 0 nm films)
